# Editorial: Nutrition and metabolic aging

**DOI:** 10.3389/fnut.2023.1191958

**Published:** 2023-04-05

**Authors:** Devin Wahl, Zachary S. Clayton

**Affiliations:** ^1^Center for Healthy Aging, Colorado State University, Fort Collins, CO, United States; ^2^Department of Health and Exercise Science, Colorado State University, Fort Collins, CO, United States; ^3^Department of Integrative Physiology, University of Colorado Boulder, Boulder, CO, United States

**Keywords:** nutrition, aging, metabolic health, calorie restriction, health span

Aging is the primary non-modifiable risk factor for most chronic and metabolic diseases including cardiovascular disorders, cancers, and neurodegeneration (1) (Jin et al.). As such, identifying interventions that slow the aging process and reduce age-associated diseases is a biomedical research priority. In recent years, several nutritional interventions have been identified that profoundly impact the aging process. These interventions can increase healthspan (the component of life that is generally healthy and devoid of chronic disorders) and lifespan. In this Research Topic, original research studies eloquently demonstrate how nutrition affects healthspan in pre-clinical models and humans (Figure 1).

**Figure 1:**
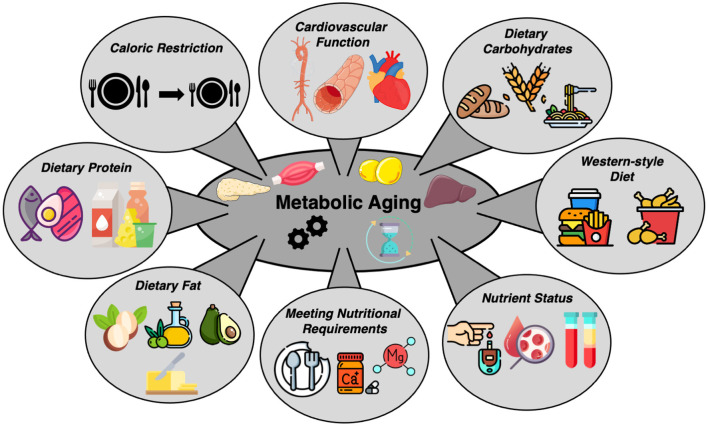
Various nutritional interventions and nutrient statuses that influence metabolic health with aging. This figure summarizes a variety of studies included in the call for papers on “Nutrition and Metabolic Aging.” This collection of papers represents studies focused on caloric restriction, consumption of a Western-style diet, macro and micronutrient intake, nutrient status and cardiovascular health, all within the context of metabolic aging.

Perhaps the most robust nutritional intervention to reduce age-associated disease and increase healthspan in pre-clinical models is calorie restriction (CR) (2), a reduction in total caloric intake by 10–50% *without malnutrition* (3). CR extends healthspan and lifespan in many pre-clinical models, and in this Research Topic, one study builds upon these findings. Teofilović et al. demonstrate that 40% CR, even if commenced later in life, improves lipid metabolism by attenuating liver fatty acid synthesis, stimulating beta-oxidation, and reducing triglyceride synthesis in rats. Interestingly, the authors find that these improvements were associated with increased liver inflammation, which may have been partly due to the older age of the rats. The results of this study underscore the need to carefully consider several factors when investigating the effects of CR in health and disease, including the age of an individual, nutritional status, and treatment duration (4).

In addition to CR, the role of dietary macronutrients (protein, carbohydrates, and fats) in modulating health has been the focus of much research (5). In this Research Topic, several studies explore the impact of macronutrient consumption in health and disease. Here, Sumi et al. investigate the effects of a low carbohydrate ketogenic diet or a high-fat, high carbohydrate diet in mice with reduced citrate synthase activity [an enzyme most often responsible for catalyzing the first reaction of the citric acid (TCA) cycle]. Interestingly, the findings of this study show that in the presence of a low-carbohydrate diet, these mice exhibit tissue-specific metabolic adaptations including decreased glycogen levels, suppression of growth pathways, and muscle atrophy. These results provide an overview of how different organs use fuel under varying energy conditions and provide insight into how metabolic disorders are related to impaired TCA cycle metabolism.

Like dietary fats and carbohydrates, dietary protein also has a profound impact on aging (6). Here, Zheng W. et al. provide insight into optimal protein consumption in older age by examining the influence of *ad-libitum* lower protein or higher protein diets on metabolic health in rats, and whether increasing dietary protein intake in older age improves health. The authors performed comprehensive assessments of behavior, the circulating metabolome, inflammation, and health, and found that shifting from lower protein consumption (in younger age) to higher protein consumption (in older age) improves select health outcomes. These results provide insight into how protein requirements change with advancing age, and the importance of protein quantity in health and disease.

Dietary fat also has an important impact on aging and age-related disease. Traditionally, chronic consumption of a high-fat Western-style diet augments age-associated disease and decreases healthspan, but many of these studies have been performed in genetically homogenous murine models. In this Research Topic, Zheng X. et al. study the effects of a chronic high-fat Western-style diet on metabolic and arterial function in outbred, genetically diverse mice—a model that has greater potential for translation to humans relative to inbred strains. The authors found that consumption of a Western-style diet increased aortic stiffness and systolic blood pressure and decreased vascular endothelial function, which are all independent predictors of cardiovascular-related morbidity and mortality (7). Importantly, these effects varied between male and female mice, emphasizing the importance of considering sex as a biological variable when assessing differences in responses to certain dietary interventions.

Li et al. build upon these findings in mice by performing a meta-analysis to study the links between saturated fatty acid metabolites and cardiovascular disease in humans. The results, which include 49 prospective studies, show that a higher abundance of circulating saturated fatty acids is associated with an increased risk of cardiometabolic disease and stroke. Similarly, Keshavarz et al. examine the impact of dairy products on non-alcoholic fatty liver disease (NAFLD). The authors use measures of liver enzymes, lipid profiles, and glycemic indexes to broadly characterize liver health and find that higher milk consumption is associated with reduced incidence of NAFLD.

This Research Topic also contains studies that provide insight into how certain dietary patterns, macronutrients, or micronutrients affect health in humans. One strong measure of healthspan in humans is glucose tolerance, and several metabolic conditions coincide with higher than average blood glucose levels (8). In this Research Topic, Ji et al. expand upon this knowledge by showing that fasting blood glucose is lower and fluctuates less in centenarians (individuals >100 years of age) when compared to younger counterparts. These results highlight the role of blood glucose in aging and emphasize the need to maintain blood glucose within a certain range to maintain healthy aging.

Finally, two interesting studies in this Research Topic demonstrate the harmful effects of undernutrition in obesity, and the importance of magnesium and calcium intake to prevent hearing loss in humans. In the first study, Sulmont-Rossé et al. performed a secondary analysis of two French surveys and found that ~23% people with a BMI over 25 are at risk of undernutrition. These results highlight the fact that more work needs to be done to comprehensively understand the role of undernourishment in affecting overweight and obesity. In the second study, Wei examined the impact of dietary magnesium and calcium intake on hearing loss in older adults and found that increased consumption of these minerals is associated with improved hearing function. These data highlight the complexity of the role of nutrition in aging and the need to consider micronutrient quantity and quality when studying metabolic health outcomes.

As the world's population ages, the burden of age-related disease magnifies. In this Research Topic, important original research studies show that healthspan can be improved with specific nutritional interventions including a reduction in caloric or saturated fat intake, optimizing dietary macronutrient consumption, increasing intake of specific minerals, and ensuring nutritional requirements are met. Together, these excellent papers provide a major step forward into our understanding of how nutrition impacts health and paves a path for future research on the role of nutrition in metabolic aging.

## Author contributions

All authors listed have made a substantial, direct, and intellectual contribution to the work and approved it for publication.
